# The impact of pharmacists-led medicines reconciliation on healthcare outcomes in secondary care: A systematic review and meta-analysis of randomized controlled trials

**DOI:** 10.1371/journal.pone.0193510

**Published:** 2018-03-28

**Authors:** Ejaz Cheema, Farah Kais Alhomoud, Amnah Shams AL-Deen Kinsara, Jomanah Alsiddik, Marwah Hassan Barnawi, Morooj Abdullah Al-Muwallad, Shatha Abdulbaset Abed, Mahmoud E. Elrggal, Mahmoud M. A. Mohamed

**Affiliations:** 1 Department of Clinical and Pharmacy Practice, College of Pharmacy, Umm-Al-Qura University, Makkah, Saudi Arabia; 2 Warwick Medical School, Gibbet Hill Campus, University of Warwick, Coventry, United Kingdom; 3 Department of Clinical and Pharmacy Practice, School of Clinical Pharmacy, University of Dammam, Dammam, Saudi Arabia; Cardiff University, UNITED KINGDOM

## Abstract

**Background:**

Adverse drug events (ADEs) impose a major clinical and cost burden on acute hospital services. It has been reported that medicines reconciliation provided by pharmacists is effective in minimizing the chances of hospital admissions related to adverse drug events.

**Objective:**

To update the previous assessment of pharmacist-led medication reconciliation by restricting the review to randomized controlled trials (RCTs) only.

**Methods:**

Six major online databases were sifted up to 30 December 2016, without inception date (Embase, Medline Ovid, PubMed, BioMed Central, Web of Science and Scopus) to assess the effect of pharmacist-led interventions on medication discrepancies, preventable adverse drug events, potential adverse drug events and healthcare utilization. The Cochrane tool was applied to evaluate the chances of bias. Meta-analysis was carried out using a random effects model.

**Results:**

From 720 articles identified on initial searching, 18 RCTs (6,038 patients) were included. The quality of the included studies was variable. Pharmacists-led interventions led to an important decrease in favour of the intervention group, with a pooled risk ratio of 42% RR 0.58 (95% CI 0.49 to 0.67) P<0.00001 in medication discrepancy. Reductions in healthcare utilization by 22% RR 0.78 (95% CI 0.61 to 1.00) P = 0.05, potential ADEs by10% RR 0.90 (95% CI 0.78 to 1.03) P = 0.65 and preventable ADEs by 27% RR 0.73 (0.22 to 2.40) P = 0.60 were not considerable.

**Conclusion:**

Pharmacists-led interventions were effective in reducing medication discrepancies. However, these interventions did not lead to a significant reduction in potential and preventable ADEs and healthcare utilization.

## Introduction

Adverse drug events (ADEs) impose a major clinical [1–2] and cost burden on acute hospital services [3]. An ADE, defined as a drug-related injury to a patient, includes physical and mental harm, or loss of function [4]. Patients are at a greater risk of experiencing an ADE through medication discrepancies to some extent during their movement within or out of hospital [5–8]. Medication discrepancies are termed as any unclear changes that have been documented in the medication lists of patients during their movement across different sites of care [9]. Around one-third of these discrepancies have the potential to harm the patients [6], which subsequently accounts for increased utilisation of healthcare resources [10–11]. Medication reconciliation has been considered as an effective strategy to minimise the risk of medication discrepancies [12] that may be potentially associated with ADEs. Medication reconciliation refers to the “process of identifying the most accurate list of all medications a patient is taking … and using this list to provide correct medications for patients anywhere within the health system” [13].

The process of medication reconciliation should account for any alterations made in the medications taken by patients and should make sure that patients or their carers have been made aware of these alterations [13]. Although, medication reconciliation does not have an impact on mortality [14], it can significantly reduce prescribing error rates [15], medication discrepancies [16–17] and unscheduled drug related visits to hospital [18].

Recent systematic reviews and meta-analysis suggest that medication reconciliation provided by pharmacists is effective in decreasing the risk of medication discrepancies [16–17, 19] adverse drug event-related hospital revisits, Emergency Department (ED) visits and admissions [20]. These studies have however; have been limited by the inclusion of observational studies [16–17, 19–20], evidence of substantial heterogeneity between the included studies [19–20] and involvement of multifaceted medication reconciliation strategies [16–17]. Furthermore, there is a need to update the current evidence on the effectiveness of pharmacist-led medication reconciliation in hospital settings. The aim of this systematic review and meta-analysis is to update the existing evaluation of the impact of pharmacist-led medication reconciliation on healthcare outcomes by restricting the review to randomized controlled trials (RCTs) only and by considering pharmacists interventions as medication reconciliation, tailored patient counselling, provision of telephonic consultation with patients post-hospital discharge and creation of post-discharge medication lists.

## Materials and methods

### Eligibility criteria

Articles were included on the basis following criteria.

#### Study designs

RCTs evaluating the effect of pharmacists based medicines reconciliation on: 1) medication discrepancies OR 2) potential adverse drug events OR 3) preventable adverse drug events OR 4) healthcare utilization post hospital discharge. Bates criteria [7] was used to categorize ADEs into preventable and potential ADEs. Although, pharmacists-led medication review has a similar approach to medicines reconciliation and some of their activities overlap with each other, the reviewers only considered activities related to medicine reconciliation in this review. The reviewers also excluded study protocols, non-RCTs, conference abstracts, non-hospital settings, non-pharmacist-intervention providers, and studies with different study outcomes.

#### Participants

It was expected that most of the RCTs eligible for inclusion in the review would include adult participants only. Therefore, participants included in the review were adults (18 years or older).

#### Comparators

RCTs were included if they had an intervention group receiving the intervention and a control group receiving standard or routine care. Usual or standard care was considered as care without the provision of medication reconciliation or provision of medication reconciliation by a healthcare professional other than the pharmacist.

#### Timing

There was no restriction on the duration of follow-up for the studies.

#### Setting

Hospital settings including all care of transitions within the hospital.

#### Language

Articles published in the English language only were included.

### Information sources

Six major online databases were sifted up to 30 December 2016, without inception date (Embase, Medline Ovid, PubMed, BioMed Central, Web of Science and Scopus). Articles were retrieved up to 30^th^ December 2016. Search terms included: “medication reconciliation”, “pharmacist”, “pharmacist-led” and “randomised controlled trials” (see Appendix 1 for the complete search strategy).

Furthermore, reference lists of all included articles were sifted to identify any relevant article. In addition, searches were also conducted in Cochrane to ensure that any eligible RCT had not been missed.

### Selection process and quality assessment

Two reviewers (MB, MM) screened the titles and abstracts of all eligible articles. Articles that qualified for inclusion were retrieved as full-text articles to finalize their inclusion. The Cochrane Risk of Bias tool [21] was used to assess the quality of included studies.

### Data collection process

Reviewer AS independently extracted data, and EC examined all extraction sheets to ensure their accuracy (see Table 1 for characteristics of included articles).

**Table 1:** Characteristic of included studies.

Author, year of publication	Study setting	Study design	Sample size	Key components of pharmacist intervention	Outcomes assessed	Intervention provider	Comparison
Stowasser et al 2002	Hospital	RCT	240	Medication liaison service-medication history confirmation with community healthcare professionals (telephone, faxing, 30 days post-follow-up)	Mortality, readmission, ED visit,	Pharmacist	Usual care (did not receive medication liaison service)
Bolas et al 2004	Hospital	RCT	164	Medication history taking, medication reconciliation, patient counselling, communication with outpatient providers	Medication discrepancies, healthcare utilization	Pharmacists	Usual care (nurses)
Nickerson et al 2005	Hospital	RCT	253	Medication reconciliation, patient counselling, communication with outpatient providers	Medication discrepancies	Pharmacists	Usual care (nurses)
Schnipper et al 2006	Hospital	RCT	178	Medication reconciliation, patient counselling, communication with outpatient providers	ADEs, healthcare utilization	Clinical pharmacists	Usual care (ward based pharmacists and nurses)
Kwan et al 2007	Hospital	RCT	464	Medication history taking, medication reconciliation	Medication discrepancies, potential ADEs	Pharmacists	Usual care (nurses)
Scullin et al 2007	Hospital	RCT	762	Integrated medicines management service admission and discharge, medicine reconciliation, inpatient medication review and counselling, telephone follow up	Length of hospital stay, readmission	Pharmacist	Usual care (did not receive integrated medicines management)
Gillespie et al 2009	Hospital	RCT	400	Medication reconciliation, patient counselling, communication with outpatient providers, medication history taking, post-discharge communication with the patients.	Healthcare utilization	Pharmacists	Usual care (no involvement of pharmacists)
Koehler et al 2009	Hospital	RCT	41	Medication reconciliation, patient counselling, communication with outpatient providers, medication history taking	Healthcare utilization	Pharmacists	Usual care (ground nursing staff)
Eggink et al 2010	Hospital	RCT	85	Medication reconciliation, patient counselling, communication with outpatient providers, medication history taking	Medication discrepancies, potential ADEs	Pharmacists	Usual care (Nurses and physicians routine activities)
Lisby et al 2010	Hospital	RCT	99	Medication reconciliation, patient counselling, communication with outpatient providers, medication history taking	Healthcare utilization, ADEs	Pharmacist	Usual care (junior physicians)
Marotti et al 2011	Hospital	RCT	357	Medication history taking, medication reconciliation	Mean no. of missed medication doses	Pharmacist	Usual care (Physicians)
Kripalani et al 2012	Hospital	RCT	862	Pharmacist-assisted medication reconciliation, tailored inpatient counselling by a pharmacist, provision of low-literacy adherence aids, and individualized telephone follow-up after discharge	Clinically important medication errors, ADEs and potential ADEs	Pharmacists	Usual care
Becerra-Camargo et al 2013	Hospital	RCT	242	Pharmacist acquired a standardised, comprehensive medication history, conducted telephone interviews with caregivers or family members, and verified with the patient if any medication changes had been made since their 24 hours in an ED.	Medication discrepancy at admission, characteristics and clinical severity of such medication discrepancies.	Pharmacists	Usual care
Hawes et al 2014	Hospital	RCT	61	Post-discharge medication reconciliation	Readmission, ED visit, readmission and or ED visit	Pharmacist	Usual care (with no pharmacist intervention)
Farris et al 2014	Hospitals	RCT	945	Created discharge care plan, telephoned patients 3–5 days post- discharge to evaluate adherence and new side effects, identified any medication-related problems and reported to the physicians.	Medication appropriateness index (MAI). Adverse events, adverse drug events and post-discharge healthcare utilization	Pharmacists	Usual care
Aag et al 2014	Hospital	RCT	201	Performed structured patient interview (to reveal all type of medicines). Also included a checklist with specific questions	Differences in the outcomes of medication reconciliation(MR) when performed by clinical pharmacists compared to nurses	Clinical pharmacists	Nurses
Farley et al 2014	Hospital	RCT	592	Minimal intervention group: clinical pharmacist case managers gave advice to patients on medication reconciliation, patient education on discharge medication. Enhanced intervention group: all the previously mentioned care along with discharge care plan and follow-up phone call from the clinical pharmacist case managers 3–5 days after discharge.	Medication discrepancies	Pharmacist	Usual care
Becerra-Camargo et al 2015	Hospital	RCT	270	Held a standardised, medication history interview with the patient during ED admission, conducted telephone interviews with caregivers or family members, reviewed medical charts, verified any changes with patients.	Percentage of potential ADEs	Pharmacists	Usual care

### Data items

A descriptive analysis of the included RCTs was carried out followed by meta-analyses. Due to expected variations in the study population of the included RCTs and their methods of measuring healthcare outcomes, a random-effects model was used (Rev-Man version 5.2). However, fixed-effect model was also used to validate the results. Heterogeneity was estimated by Cochrane’s test and further analysed using I2.

### Outcomes and prioritisation

Four outcomes were assessed with equal priority in the review, and these were:

- Medication discrepancies, termed as any unclear changes that have been documented in the medication lists of patients during their movement across different sites of care [9].
- Potential adverse drug events, termed as adverse drug events with potential to cause injury [7].
- Preventable adverse drug events, termed as adverse drug events that were preventable [7].
- Healthcare utilization, termed as utilization of healthcare resources through drug-related Emergency Department visits or hospital readmissions [10].

## Results

**Fig 1:**
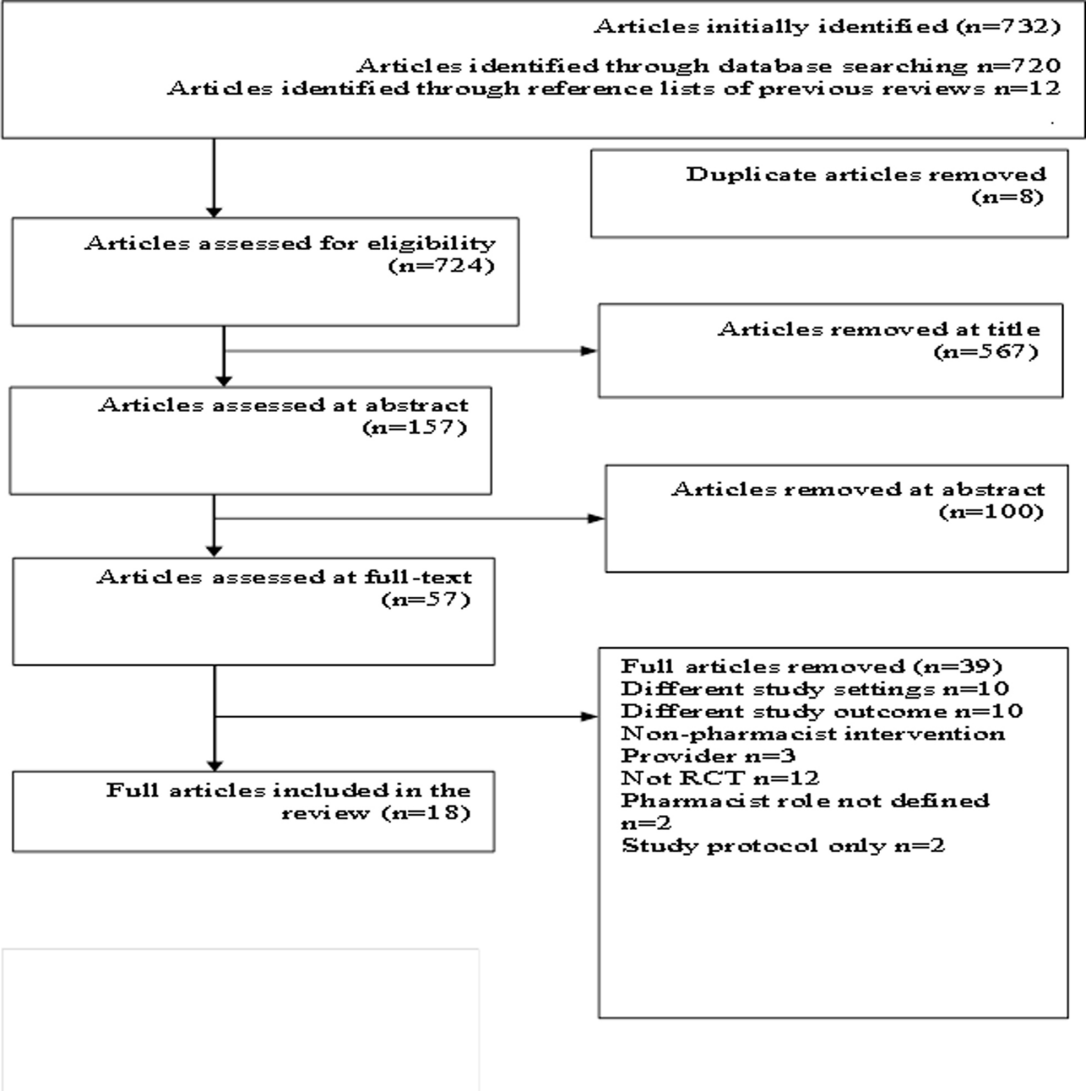
Prisma flow diagram representing the selection process of articles included in the review.

A total of 732 articles were identified initially (see Fig 1 for Prisma flow diagram) that included 720 from electronic databases and 12 from reference lists of previous reviews. Of these 732 articles, eight duplicates were removed and remaining 724 articles were assessed for eligibility. After the removal of 567 articles with irrelevant titles, 157 articles were assessed at abstract level. After the removal of 100 abstracts that did not meet the eligibility criteria, 57 full-text articles were assessed for further eligibility. Of these 57 full-text articles, 39 failed to meet the eligibility criteria. Reasons for their removal included: not RCTs, different study settings, intervention not provided by pharmacists, different study outcome, studies not defining pharmacist roles and studies with published protocol only. Finally, 18 RCTs contributed to the systematic review [22–39].

### Study quality

**Fig 2:**
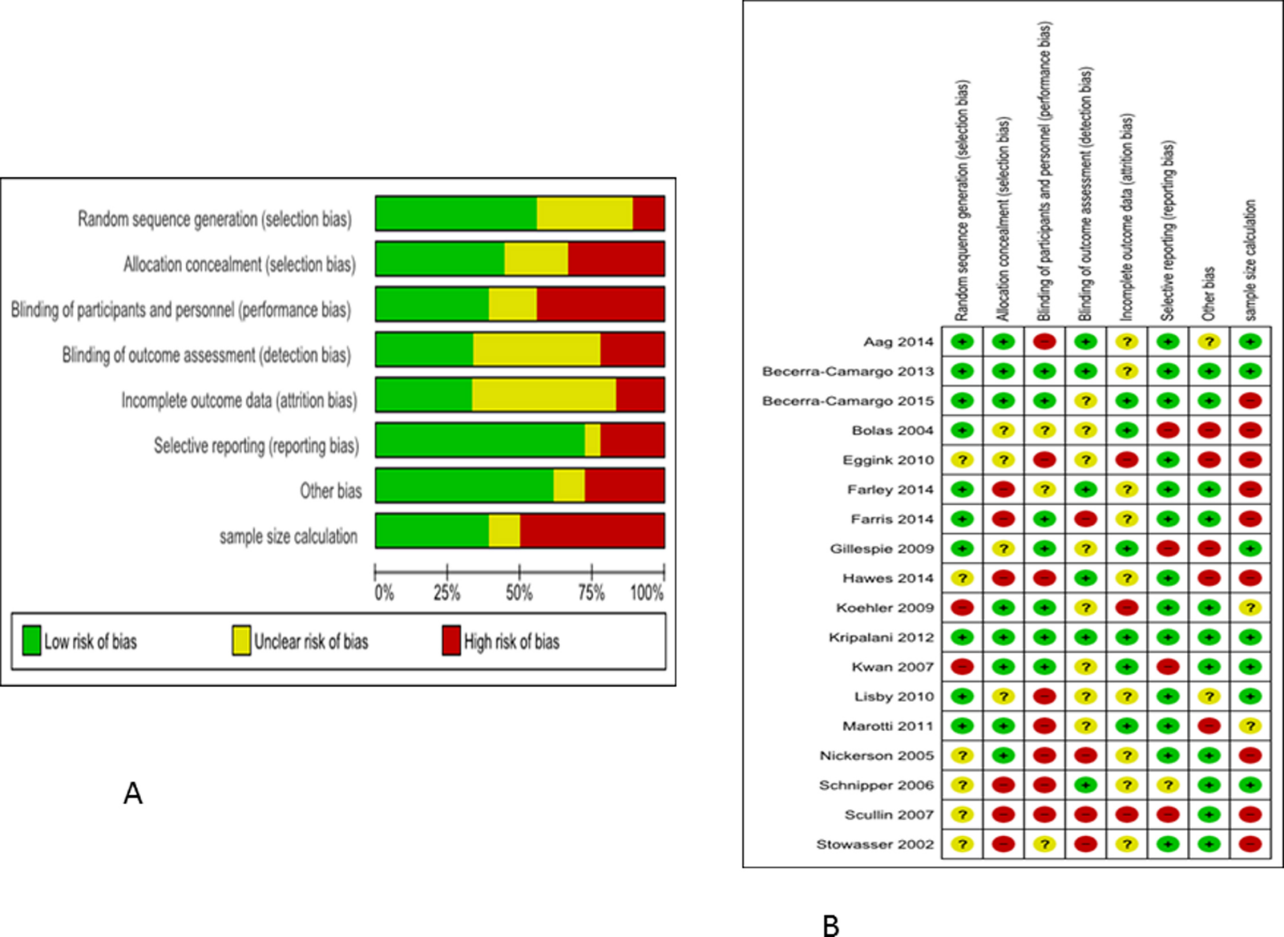
(a) Risk of bias graph: review authors' judgements about each risk of bias item presented as percentages across all included studies. 2 (b) Risk of bias summary: review authors' judgements about each risk of bias item for each included study.

Only eight (44%) of the 18 articles reported allocation concealment [22–24, 31–33, 35–36]. It was not clear in the remaining 10 articles whether they had used allocation concealment (see Fig 2A and 2B). Only six (33%) articles reported details of missing outcome data [24–25, 29, 32–33, 35] while only seven (39%) articles reported power calculations [22–23, 29, 32–34, 37].

### Study characteristics

All 18 included articles were RCTs that were conducted in hospital settings. Included RCTs were conducted in the United States [27–28, 30–32, 37], Canada [33, 36], Australia [35, 39], Colombia [23–24], Ireland [25, 38], Norway [22], Netherlands [26], Sweden [29] and Denmark [34].The RCTs included 6,038 patients, with a population range of 41 [31] to 945 [28]. All 18 articles had an intervention group that received one or more of the pharmacists-led interventions including: medication history taking, medication reconciliation, patient counselling, creation of post-discharge medication lists compared with a control group receiving usual care (see Table 1 for characteristics of included studies).

### Impact of pharmacist interventions on outcome measures

Of the 18 RCT, 10 were included in the meta-analysis [23, 26, 27–28, 31–34, 37–38]. All 10 RCTs included in the meta-analysis employed three similar pharmacist-led interventions: medication reconciliation, tailored patient counselling and provision of telephonic advice to patients post-hospital discharge. Absence of quantitative study outcome data on the reviewed study outcomes for example in studies [22, 24, 35] and use of multidisciplinary interventions including general practitioners and community pharmacists, for example in study [39] were reasons to remove the remaining eight RCTs from meta-analysis [22, 24–25, 29–30, 35–36, 39].

#### Medication discrepancy

**Fig 3:**
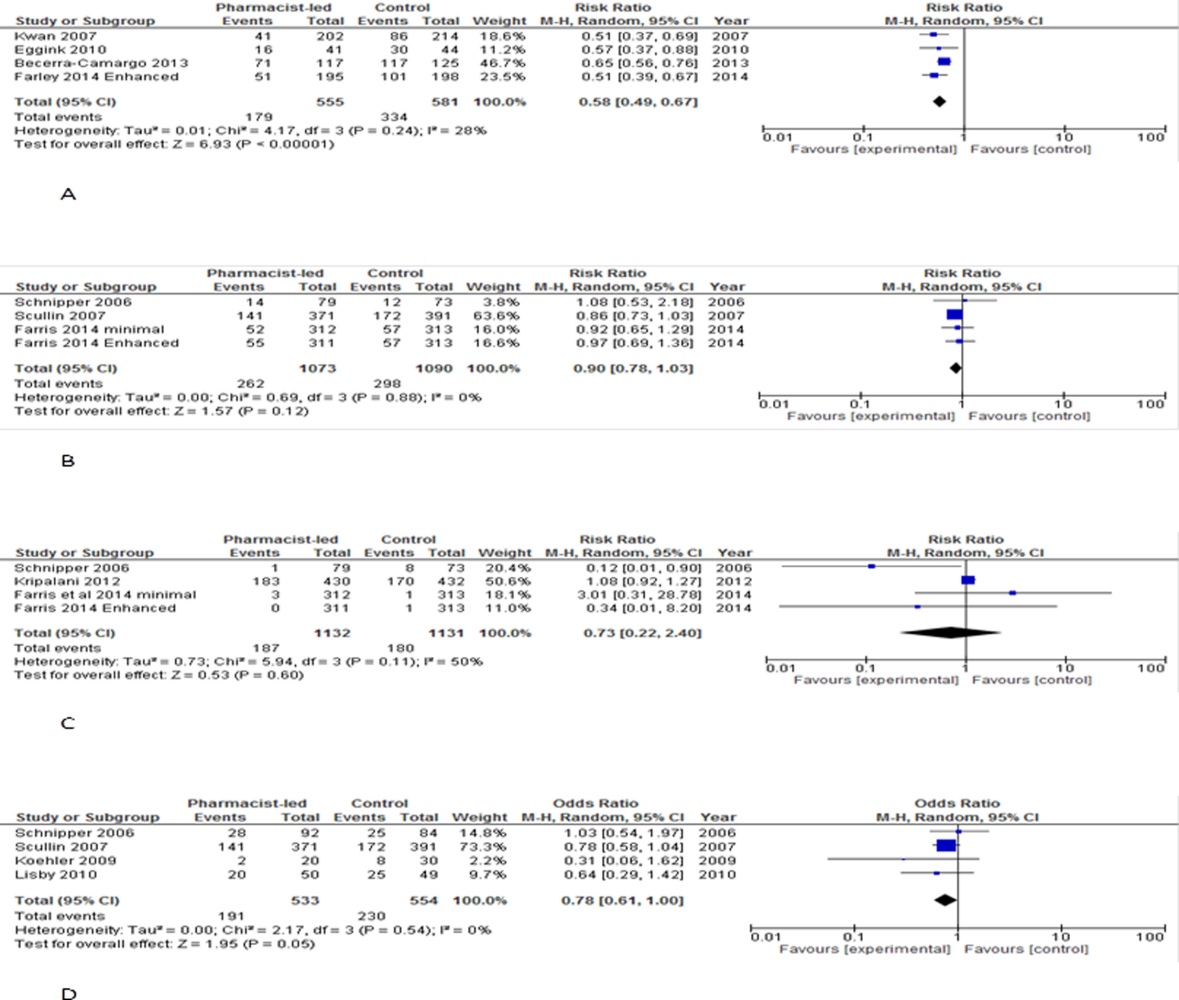
Forest plot comparisons of experimental (intervention) vs. control groups in four studies for medication discrepancy (A) three studies for potential ADEs (B) three studies for preventable ADEs (C) and four studies for healthcare utilization post-hospital discharge (D). Pharmacists-led interventions included medicine reconciliation and tailored patient counselling post hospital discharge. Farley [27] and Farris [28] used two tiers of pharmacist interventions: enhanced and minimal.

Four of the seven RCTs that reported data on medication discrepancy were included in the meta-analysis [23, 26–27, 33]. Meta-analysis of data from four RCTs (1,102 patients) reported an important reduction in favour of the intervention group with a pooled risk ratio of 42% RR 0.58 (95% CI 0.49 to 0.67) P<0.00001. No difference was reported between the effects of random and fixed-effect model meta-analysis. Heterogeneity among the RCTs was low (χ2 = 4.17, d.f. = 3, P = 0.24, I2 = 28%; Fig 3A).

#### Potential ADEs

Three of the four RCTs that reported data on potential ADEs were included in the meta-analysis [28, 37–38]. Meta-analysis of these RCTs (1,885 patients) showed a small reduction in favour of the intervention group, with a pooled risk ratio of 10% RR 0.90 (95% CI 0.78 to 1.03) P = 0.65. There was no heterogeneity among the RCT (χ2 = 0.69, d.f. = 3, P = 0.88, I2 = 0%; Fig 3B).

#### Preventable ADEs

Three studies reported data on preventable ADEs and were included in the meta-analysis [28, 32, 37]. Meta-analysis of these three RCTs (1,985 patients) showed a small reduction in favour of the intervention group, with a pooled risk ratio of 27% RR 0.73 (95% CI 0.22 to 2.44) P = 0.60. Heterogeneity among the RCTs was low to moderate (χ2 = 5.94, d.f. = 3, P = 0.11, I2 = 50%; Fig 3C).

#### Healthcare utilization post-hospital discharge

Of the eight studies that reported data on healthcare utilisation, four were included in the meta-analysis [31, 34, 37–38]. Meta-analysis of four studies (1,080 patients) showed a non-significant reduction in favour of the intervention group, with a pooled risk ratio of 22% RR 0.78 (95% CI 0.61 to 1.00) P = 0.05. No difference was reported between the effects of random and fixed-effect model meta-analysis. There was no heterogeneity among the RCTs (χ2 = 2.17, d.f. = 3, P = 0.54, I2 = 0%; Fig 3D).

### Sensitivity analysis for the study outcomes

A step-wise exclusion of studies included in the meta-analysis did not make any significant difference in the results of any outcomes.

## Discussion

The findings of this systematic review show that, compared with usual care, active interventions by pharmacists including medication reconciliation, tailored patient counselling, and provision of telephonic consultation with patients following hospital discharge were associated with clinically important reduction in medication discrepancies. There was a non-significant reduction in favour of the intervention group for potential and preventable ADEs and healthcare utilization.

Previous reviews have assessed the impact of pharmacist interventions on clinical outcomes across various hospital transitions by including observational studies [16–17, 19–20] and by involving multifaceted interventions [16–17]. This study has updated the existing evaluation of the impact of pharmacist-led medication reconciliation on healthcare outcomes by including a further seven RCTs that were not referenced in the last published review conducted by Mekonnen et al [20].

The findings of this review suggest that interventions provided by pharmacists led to important reductions in medication discrepancies across a wide range of international geographical regions from North and South America to Europe and Australia. These findings are similar to the findings of the previous review [20] that reported important reductions in medication discrepancies as a result of pharmacist interventions. Medication discrepancies can occur at either hospital admission stage or at discharge [40–41] and can contribute to potentially harmful adverse events. A study involving patients 65 years and older reported that 14.3% of the patients who had one or more medication discrepancy post-hospital discharge were re-admitted to the hospital after one month compared to 6.1% of patients with no discrepancy [42]. The decrease in medication discrepancies reported in this review suggests the significance of collecting accurate and complete medication histories in reducing the incidence of potential adverse events.

Completion of accurate and comprehensive medication histories across all hospital transitions is also helpful in reducing healthcare utilizations following hospital discharge as reported in this review. All eight studies that assessed the impact of pharmacist-led interventions on healthcare utilization post-hospital discharge reported reduction in healthcare utility by reducing hospital readmissions and Emergency Department visits. These findings are in contrast to the findings of a previous review by Mueller et al [16], where only two of the eight studies reported reduction in healthcare utilization following hospital discharge. However, that review used multifaceted interventions related to information technology, pharmacist-based and other types. A study conducted in Sweden that included 368 patients reported that patients who received pharmacist interventions experienced an overall 16% reduction in hospital visits including a 47% reduction in visits to the Emergency Department [29]. Furthermore, pharmacist interventions were also associated with an 80% reduction in drug-related readmissions.

The evidence presented in this review that is consistent with previous reviews [16–17, 19–20] suggests the important role of pharmacists in taking accurate medication history across various hospital transitions. Pharmacists’ increased knowledge and acquaintance with the medications allows them to extend support to other healthcare professionals including doctors and nurses by acquiring precise and complete medication histories from the patients [43–44]. For example, a study by Kwan et al [33] reported that pharmacists who interviewed patients as part of medication reconciliation identified more medications per patient as compared to doctors and nurses. However, owing to the extensive involvement of pharmacists in other pharmacy services, they may not be available to conduct medicine reconciliation at all times. In such busy times, senior pharmacy technicians along with nurses should continue to perform the process of medicines reconciliation.

There were limitations in this review. The reviewers did not include unindexed and unpublished research. Studies were of variable quality. Within our efforts to limit heterogeneity, we included studies in the meta-analysis that had similar study design and used similar interventions, yet low to moderate heterogeneity between the studies included for the assessment of some of the outcomes suggests that medication reconciliation based interventions are complex. Some of the studies used a coordinated model of care that may explain the complexity of these interventions. For example, a study by Stowasser et al [39] employed a medication liaison service where clinical pharmacists, in consultation with general practitioners, community pharmacists and hospital staff, prepared a comprehensive medication list at both hospital admission and prior to hospital discharge. Patients who received the medication liaison service experienced better patient outcomes including a significant reduction in hospital readmissions and healthcare professional visits per patient.

## Conclusions

Pharmacists-led interventions were effective in reducing medication discrepancies. However, these interventions did not lead to a significant reduction in potential and preventable ADEs and healthcare utilization. The quality of studies included in this review was variable and therefore the findings of this review must be interpreted with caution. Future studies are required to evaluate the impact and sustainability of pharmacist-led interventions on healthcare outcomes in the long term.

## Appendix 1: Search strategies used in the major electronic databases

**Embase**:

1. Medicine reconciliation.mp.
2. Medicine reconciliation Or exp medicines reconciliation/
3. 1 or 2
4. Exp pharmaceutical care/ or exp pharmacy/ or exp pharmacist/
5. pharmac*.mp.
6. 4 or 5
7. Exp pharmacist/ or pharmacis*.mp.
8. Intervention*.mp.
9. 3 and 6 and 7 and 8
10. limit 9 to (English language and randomized controlled trial and (adult <18 to 64 years> or aged <65+ years>)

**Medline Ovid**:

1. Medicine reconciliation.mp.
2. Medicine reconciliation Or exp medicines reconciliation/
3. 1 or 2
4. Exp pharmaceutical care/ or exp pharmacy/ or exp pharmacist/
5. pharmac*.mp.
6. 4 or 5
7. Exp pharmacist/ or pharmacis*.mp.
8. Intervention*.mp.
9. 3 and 6 and 7 and 8
10. limit 9 to (English language and randomized controlled trial and (adult <18 to 64 years> or aged <65+ years>)

**Web of Science**:

1. Pharmacist OR pharmacists OR pharmaceutical AND
2. Medicines reconciliation

**Pubmed**:

1. Pharmacists OR pharmacist interventions
2. Medicines reconciliation
3. RCTs

**Scopus**:

Pharmacist OR pharmacists OR pharmaceutical AND
1. Medicines reconciliation AND
RCTs

**Biomed Central**:

1. Pharmacists interventions ALL WORDS
2. Medicines reconciliation AND
3. Randomised controlled trial AND

## Supporting information

S1 Checklist Revised PRISMA-2009-checklist-MS-word.doc. (DOC)
